# Does Nutrient Availability and Larval Competition Alter Chikungunya Virus Infection in the Mosquito *Aedes albopictus*?

**DOI:** 10.3390/v17050613

**Published:** 2025-04-25

**Authors:** Maria Eduarda Barreto Resck, Nildimar Alves Honório, Barry Wilmer Alto

**Affiliations:** 1 Laboratório das Interações Vírus-Hospedeiros—LIVH, Instituto Oswaldo Cruz/Fiocruz, Rio de Janeiro 21040-900, Brazil; 2 Núcleo Operacional Sentinela de Mosquitos Vetores-Nosmove/Fiocruz, Rio de Janeiro 21040-900, Brazil; 3 Florida Medical Entomology Laboratory—FMEL, Institute of Food and Agricultural Sciences, University of Florida, Vero Beach, FL 32962, USA

**Keywords:** *Aedes albopictus*, intraspecific larval competition, nutrient availability, susceptibility to infection, Chikungunya virus

## Abstract

*Aedes albopictus* is a mosquito that has spread rapidly in the United States and is considered an important vector for arbovirus transmission to humans in several countries. Larval interactions and environmental conditions can influence mosquitoes and their ability to transmit pathogens as adults. We investigated whether intraspecific larval competition among *Ae. albopictus* mosquitoes from Florida, combined with varying food availability, affects vector competence for Chikungunya virus (CHIKV). We reared larvae under four competition treatment densities and two food levels. Measurements were taken for larval development duration, survival rate, and female wing length. Mosquitoes from each treatment group were orally challenged with CHIKV. Our results showed that development time was longer for both female and male *Ae. albopictus* under high-competition conditions and appeared as the most important factor, followed by survivorship. Survival rates were highest under low-density conditions compared to those reared under high-density conditions. Mosquitoes reared with a low amount of food had the lowest survivorship and longest development times compared to those provided with high food levels. Our results also showed susceptibility infection and disseminated infection of CHIKV was influenced by an interaction of density and food availability. Mosquitoes from the high-food, high-density treatment group exhibited lower CHIKV infection and dissemination rates compared to other treatment combinations. These findings highlight the role of larval competition and nutritional stress during immature stages in shaping adult mosquito traits, with important epidemiological implications for CHIKV transmission.

## 1. Introduction

Native to Asia and introduced in North America during the 1980s, *Aedes albopictus* is a mosquito that has spread rapidly in the southern and northern regions of the United States [1,2]. The success of *Ae. albopictus* introduction in several continents such as North America, South America [3], Africa, Europe [4], and the Pacific Islands region is due to its generalized habitat and food requirements, desiccation-resistant eggs, adaptability to different climatic conditions, and an ability to live in human-dominated habitats [1,5,6]. 

As an introduced species, it is of utmost importance to understand the ecology of *Ae. albopictus* and how biotic interactions such as predation, competition, and mutualism affect this species and other members of its community [7,8,9]. The effects of competition during the aquatic immature stages can carry over and alter phenotypic traits of adults and intra- and interspecific interactions, including susceptibility to arbovirus infection and transmission [10]. Field and laboratory studies of interspecific competition among larvae, and reproductive interference (i.e., satyrization) among adults, of *Ae. albopictus* and *Ae. aegypti* demonstrated that *Ae. albopictus* has a competitive advantage with low-nutrient conditions [11,12]. *Ae. albopictus*’ competitive advantage, especially in low-nutrient environments, is thought to contribute to the displacement of *Ae. aegypti* in many U.S. regions. However, coexistence is possible depending on seasonal environmental differences in their habitats [13,14,15].

Previous studies have shown that *Ae. albopictus* larval competition can affect traits of adult mosquitoes, such as morphology [16,17,18,19], lifespan [17,19,20], host-seeking behavior [21,22], and vector competence for arboviral pathogens such as dengue and Sindbis viruses [16,18,20]. Many of these factors contribute to vectorial capacity, an index of risk of pathogen transmission. For example, competition-induced changes in growth and associated adult size influence the adult lifespan, a parameter that strongly contributes to vectorial capacity [23,24]. 

Vector capacity refers to the overall efficiency of a vector population to transmit a pathogen in nature, which includes factors such as vector competence, vector density, biting rate, and lifespan. A laboratory measure commonly used for determining the potential of a vector to become infected and transmit a pathogen is called vector competence. In this context, we define it as susceptibility to infection, replication, and transmission of an arbovirus. For transmission to occur, an arbovirus is usually acquired from an infected host during blood feeding. Blood is deposited in the mosquito midgut where initial infection occurs. Subsequently, the viral infection can spread (disseminate) beyond the midgut to secondary organs, including the salivary glands and reproductive organs, making arbovirus transmission possible during blood feeding and oviposition [25,26]. 

Chikungunya virus (CHIKV), a mosquito-borne illness, has spread rapidly from tropical regions to subtropical areas and the Western Hemisphere. This arbovirus is transmitted by invasive *Stegomyia* mosquitoes, and urban outbreaks have been reported worldwide, especially in regions where these mosquitoes are prevalent [5,27,28,29,30]. The rapid global spread of CHIKV infections was characterized by a high rate of illness, neurological complications, and atypical cases of Guillain–Barré syndrome, transverse myelitis, microcephaly, and meningitis, posing a significant threat to public health worldwide [31]. The adaptation of CHIKV to *Ae. albopictus* has played a significant role in the global spread of CHIKV. Genetic analysis of complete viral genomes has identified distinct adaptive variants in at least three instances, suggesting that these mutations have conferred a selective advantage for CHIKV transmission by *Ae. albopictus* [32,33,34,35,36].

In this study, we aimed to investigate whether larval competition among Florida *Ae. albopictus* mosquitoes influences the vector competence for CHIKV. We test the hypothesis that nutrient limitation and larval competition decreases population growth correlates and increases susceptibility to infection, disseminated infection, and transmission of CHIKV by adult mosquitoes.

## 2. Materials and Methods

### 2.1. Ethics Statement

The Asian Chikungunya virus strain used in this study was previously isolated from a human patient in the British Virgin Islands in 2013 (GenBank accession KJ451624), obtained from the Centers for Disease Control and Prevention’s Arboviral Diseases Branch.

### 2.2. Mosquitoes

*Ae. albopictus* mosquitoes were obtained from laboratory colonies established through collections of larvae and pupae from containers in Vero Beach, FL (latitude, 27.5876; longitude, −80.3714 in decimal degrees) adjacent to the campus of the University of Florida, Florida Medical Entomology Laboratory (UF-FMEL). Approximately 200 field-collected larvae and pupae were used to establish a low-passage lab colony of *Ae. albopictus*. The mosquitoes were maintained in cages (31 width × 32 length × 33 cm height) in an insectary (28 °C and a 12 h light/12 h dark cycle photo regime) and provided with 10% sucrose solution through cotton wicks and weekly blood meals from chickens (IACUC protocol 201003892) at the UF-FMEL. A water-filled plastic cup with a damp paper towel lining the inside of the cup served as an oviposition substrate for females within cages (300 mosquitoes per cage). The progeny eggs from *Ae. albopictus* (F9 generation) were hatched in deoxygenated tap water in an Erlenmeyer flask for 60 min using a vacuum to induce synchronous hatching. Newly hatched larvae were placed in rearing pans with 1 L tap water and fed a diet of larval food consisting of equal parts of *Saccharomyces cerevisiae* (brewer’s yeast) and bovine lactalbumin.

### 2.3. Nutrient Availability and Intraspecific Competition

One day post-hatching, the first instar larvae were rinsed from food and water and allocated to four density treatments (30, 60, 120, and 180 larvae) with 5-fold replication. Larvae were placed in clear plastic containers (with lids) along with 400 mL tap water and plant and insect detritus as basal resources for microbial growth and larval food.

Two larval food treatments were used: 1—low food level, consisting of 1.31 g of senescent oak leaves (*Quercus virginiana*) and 0.08 g of commercially available dried crickets (*Acheta domesticus*, Fluker’s Freeze-Dried Crickets, Port Allen, LA, USA); 2—high food level, which consisted of twice the amounts of oak leaves and dried crickets used for low food levels. Treatment containers were maintained in a bioroom set at 28 °C with a 12 h light/12 h dark cycle photo regime. Pupae were removed from the containers and stored in groups of 5 in 37 mL tap water-filled vials and covered with cotton until emergence as adults. Treatment containers and vials with pupae were monitored daily until all individuals emerged as adults or died as immatures.

Three correlates of population growth were measured to quantify the effects of intraspecific competition on mosquito performance: adult size, development time (time from egg hatch to adulthood), and survivorship to adulthood. For adult size, a single wing from each mosquito was dissected and mounted on glass microscope slides and wing length was measured from alula to wing tip using computer imaging software (IMT i-Solution lite, version 10, Princeton, NJ, USA). We also measured mean female and male dry mass of adults using a microbalance (Orion Cahn C-33, Thermo Scientific, Wilmington, DE, USA). Survivorship was calculated as the number of adults (females and males) divided by the total number of original larvae, expressed as a percentage. These measurements were used to calculate lambda (λ′), which estimates the finite rate of population increase [37], and were determined for each treatment replicate:

$$\lambda^{\prime }=\;\exp\left(r^{\prime }\right)=\exp\;\frac{\ln\;\left[\left(1/N_{o}\right)\sum_{x}\;A_{x}\;f\left(w_{x}\right)\right]}{D\;+\left[\sum_{x}\times A_{x}\;f\left(w_{x}\right)\;/\sum_{x}\;A_{x}\;f\left(w_{x}\right)\right]}$$

where *N*_0_ is the initial number of females (assumed to be 50% of a cohort), *A_x_* is the number of females eclosing on day *x*, *w_x_* is the mean mass of females eclosing on day *x*, and *f*(*w_x_*) is a function relating number of eggs to female mass. *D* is the time from adult eclosion to reproduction, assumed to be 14 d for *Ae. albopictus* [38]. A regression relating adult female dry mass to fecundity for *Ae. albopictus* was obtained by Lounibos et al. [39]:

*f*(*w_x_*) = 19.5 + 152.7 *w_x_*

where r^2^ = (sqrt[r^2^_1_] × sqrt[r^2^_2_])^2^ = 0.573.

### 2.4. Chikungunya Virus Infection

Virological procedures were performed at the UF-FMEL biosafety level (BSL) 3 facility and followed the BSL-3 standard operating procedures currently established. For virus suspension preparation, African green monkey (Vero) cell monolayers were inoculated with diluted CHIKV stock at a multiplicity of infection (i.e., number of viruses per cell) of 0.1 followed by a one-hour incubation at 37 °C and a 5% carbon dioxide atmosphere. After the inoculation procedure, each flask received 20 mL of medium (M199 medium supplemented with 10% fetal bovine serum, 2% penicillin/streptomycin, and 2% nystatin (Mycostatin)) and maintained at 37 °C and 5% carbon dioxide for a 3-day incubation period based on cytopathology.

Nine-to-fourteen-day-old adult females resulting from each treatment replicate were fed commercially purchased defibrinated bovine blood (HemoStat Laboratories, Dixon, CA, USA) containing freshly propagated CHIKV (Asian genotype, GenBank accession: KJ451624, isolated from the serum of an infected human in the British Virgin Islands in 2013) warmed to 37 °C and offered to mosquitoes through an artificial membrane feeding system (Hemotek, Lancashire, UK) for 1 h at 28 °C.

Aliquots of 1 mL of CHIKV-infected blood were taken before and after each blood feeding trial and stored in 2 mL cryogenic vials (Millipore Sigma, Burlington, MA, USA) at −80 °C for determination of viral titer of infected blood. The CHIKV titer in blood meals was 8.4 log_10_ plaque-forming unit equivalents per mL (PFUe/mL).

### 2.5. Chikungunya Virus Dissemination and Transmission

Following feeding trials, females were anesthetized with carbon dioxide and kept cool within a metal tray set on ice. Mosquitoes were sorted and fully engorged females were transferred to cages (0.47 L food containers with mesh lids) and held for a 7-day incubation period until sample collections were tested to determine susceptibility to infection, disseminated infection, and transmission rates. To determine susceptibility to viral infection and disseminated infection, each mosquito body and legs, respectively, were homogenized (TissueLyser II sample disruptor; Qiagen, Germantown, MD, USA) in medium supplemented with fetal bovine serum and centrifuged before viral RNA isolation and detection by PCR. For transmission assays, females were deprived of sucrose, but not water, for 24 h and transferred to 37 mL plastic tubes (height x diameter: 8 by 3 cm) with a removable mesh lid, containing cationic paper (Q) “Q-paper” (1 cm in diameter) [40] soaked with honey dyed with blue food coloring (McCormick, Hunt Valley, MD, USA) for female salivation, as previously described in Honório et al. [41]. However, due to the low numbers of females that fed on honey, we were not able to provide a comprehensive assessment of treatment effects on transmission. Thus, the remainder of the infection portion of the study focused on susceptibility to infection and disseminated infection of CHIKV. The latter measurement is a prerequisite for transmission and represents an advanced state of infection.

### 2.6. Viral Nucleic Acid Extraction and Quantitative RT-PCR

After dissecting and trituration of mosquito bodies and legs, RNA isolation was performed on individual samples. Briefly, 140 μL of body and leg sample homogenates were processed using the QIAamp Viral RNA Mini Kit (Qiagen, Valencia, CA, USA) and eluted in 60 μL of buffer, according to the manufacturer’s protocol. CHIKV viral RNA was detected and quantified from individual samples by RT-qPCR, using the SuperScript III Platinum RT-qPCR Kit (Invitrogen, Carlsbad, CA, USA) in a CFX96 thermocycler (Bio-Rad Laboratories, Hercules, CA, USA). Each reaction comprised of a master mix containing 10 μL of 2× Reaction Mix, 2.2 μL of diethyl pyrocarbonate (DEPC) treated water, 1.0 μL of forward primer (10 μM), 1.0 μL of reverse primer (10 μM), 0.4 μL SuperScript™ III RT/Platinum™ Taq Mix, 0.4 μL of probe, and 5.0 μL of viral RNA template (25% of reaction volume) for a total volume of 20 μL. Negative controls consisted of a sham viral RNA template of DEPC-treated water or dilute stock virus, each using a volume of 5.0 μL. Each mosquito sample and controls were tested in duplicate. Each sample with a quantification cycle (Cq) value of <35 was determined as positive. The thermocycling conditions were as follows: 50 °C for 30 min, 94 °C for 2 min, 39 cycles at 94 °C for 10 s and 60 °C for 1 min, and 50 °C for 30 s. The following sequences represent primers designed to target a nonstructural polyprotein gene (accession ID of transcript, KU365292.1): forward, 5′-GTACGGAAGGTAAACTGGTATGG-3′; reverse, 5′-TCCACCTCCCACTCCTTAAT-3′. The probe sequence was 5′-/56-FAM/TGCAGAACCC ACCGAAAGGAAACT/3BHQ_1/-3′ (Integrated DNA Technologies, Coralville, IA, USA). These primers and probe have been used successfully in previous studies assessing vector competence of mosquitoes for CHIKV [41,42,43]. Viral titer quantification was determined using a standard curve with serial dilutions of CHIKV stock, in parallel with titration by plaque assays of the same virus dilutions, expressed as plaque-forming unit equivalents (pfue)/mL [41,44].

**Figure 1:**
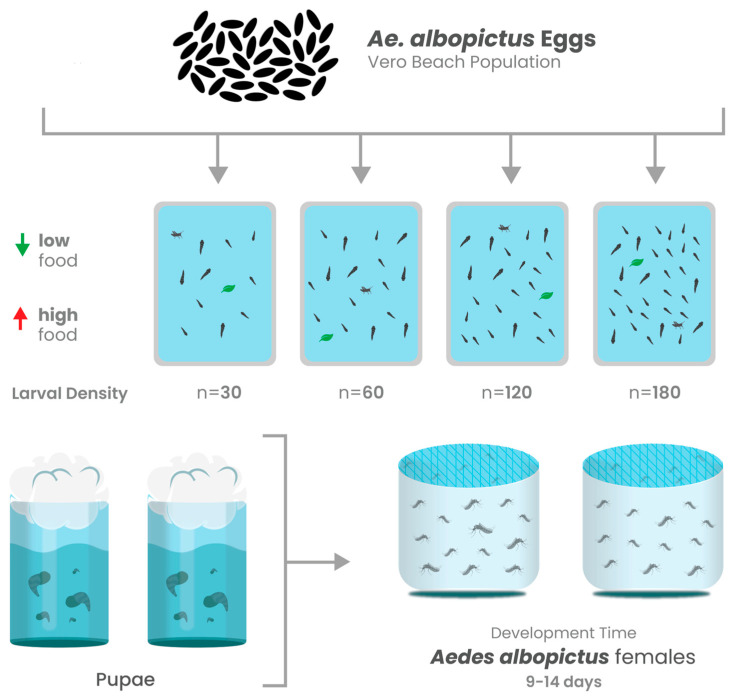

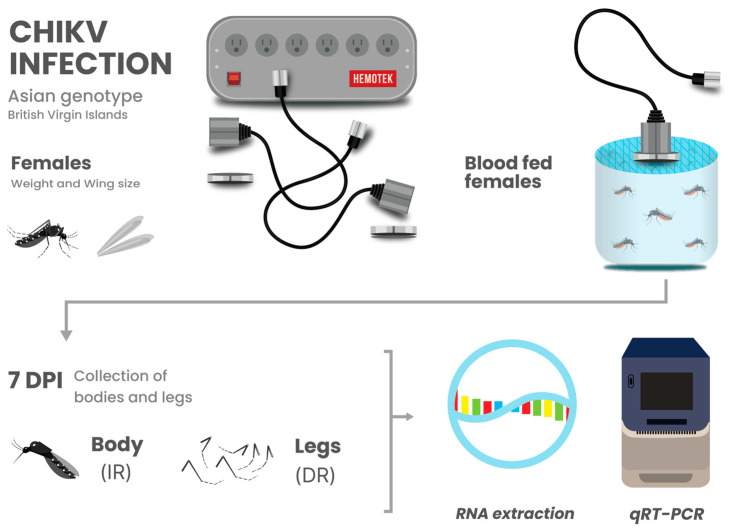
Schematic overview of experimental design. Mosquito population growth correlates (development time, adult eclosion rates, and adult size) were determined for mosquitoes from each experimental treatment. Adult female mosquitoes from each treatment were orally challenged with CHIKV infected blood to determine infection rate (IR), dissemination rate (DR), and whether larval competition and nutrition altered the course of infection.

Infection rate was determined by the number of females with CHIKV RNA-positive bodies from the total number that fed on the infectious blood meal. Disseminated infection rate was determined by the number of females with infected bodies that had CHIKV RNA-positive legs [41,43]. Figure 1 provides a detailed schematic overview of the experimental design.

### 2.7. Statistical Analysis

Individual Multivariate Analysis of Variances (MANOVAs) were used to determine the effects of intraspecific larval competition (larval density) and nutrition treatments (food level) on three population growth correlates: development time, survivorship to adulthood, and adult size of males and females. Standardized canonical coefficients (SCCs) were used to determine the relative contribution of each population growth correlate (adult size, survivorship, and developmental time) to significant multivariate effects as well as their relationship to each other (e.g., positive or negative association; Scheiner [45]; SAS Institute 2002). Treatment effects on the finite rate of increase (lambda λ′) were analyzed using ANOVA. When significant effects were detected, post hoc procedures used pairwise comparisons of means adjusted for α = 0.05 (Tukey–Kramer adjustment, PROC GLM, SAS 9.22). Competitive treatment effects on body and leg viral titers of *Ae. albopictus* were analyzed by two-way ANOVA. Separate logistic regression analyses were used to determine the effect of larval competition (larval density) and nutrition (food level) on susceptibility to CHIKV infection and disseminated infection.

## 3. Results

### 3.1. Competition

Population growth (lambda λ′) analysis showed that for all densities of *Ae. albopictus*, all mean values of lambda λ′ were >1, suggesting that cohorts of mosquitoes were increasing under all conditions analyzed (Table 1). We tested pairwise differences for all densities, food levels, or interaction of both treatment effects using the parametric Tukey test. However, no statistically significant differences were detected among densities (*p* > 0.48), food levels (*p* = 0.67), or their interaction combinations (all *p* > 0.64).

**Figure 2:**
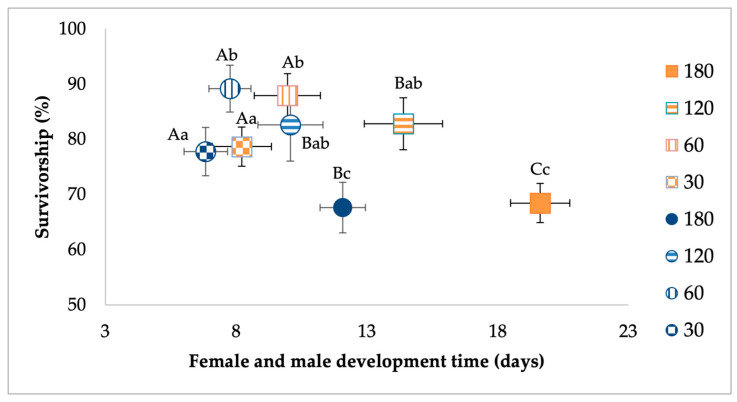
Analysis of the effects of larval density treatment on survivorship and development time of *Ae. albopictus* males (in circles) and females (in squares). Bivariate least-squares means (±standard error) are shown for dependent variables contributing the most to significant multivariate treatment effects. Means followed by different letters show significant differences (development time, capital letters; survivorship, lower-case letters).

MANOVA analysis for females showed a significant effect of larval density. The food treatment and interaction between food and density were not significant (Table 2). For the larval density effect, standardized canonical coefficients (SCCs) showed that the development time contributed the most followed by survivorship (Table 2). Pairwise contrasts for development showed that higher density treatments were associated with longer development time (Figure 2). Pairwise contrasts for survivorship showed the highest rates of eclosion to adulthood were observed in treatments with low density of 60 larvae compared to the highest-density treatment of 180 larvae (Figure 2).

**Figure 3:**
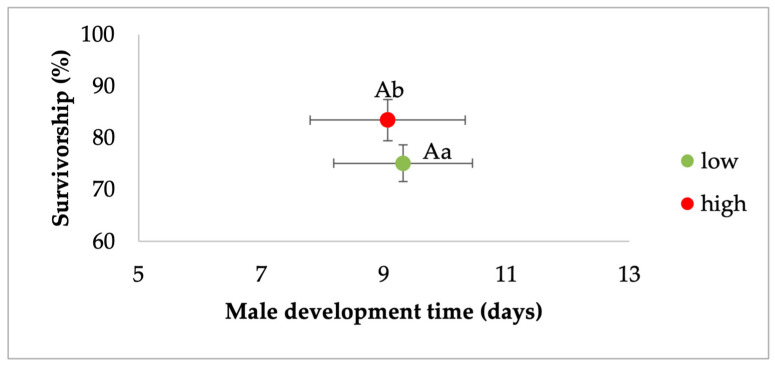
Analysis of the effects of food treatment on survivorship and development time of *Ae. albopictus* males. Bivariate least-squares means (±standard error) are shown for the dependent variables which had the greatest contribution to the multivariate effect. Means followed by different letters show significant differences (development time, capital letters, survivorship, lower-case letters).

MANOVA for *Ae. albopictus* males showed a significant effect of larval density and food, but not their interaction (Table 3). For the density effect, standardized canonical coefficients (SCCs) showed that the development time contributed the most followed by survivorship (Table 3). For the food effect, SCCs showed that development time and survivorship had a similar and high contribution. Pairwise contrasts for development showed that higher-density treatments were associated with longer development time (Figure 2). Pairwise contrasts for survivorship showed the highest rates of eclosion to adulthood for a low density of larvae compared to the highest-density treatments (Figure 2). For the food effect, mosquitoes from relatively low-nutrient conditions had lower survivorship and longer development times compared to the high-food conditions (Figure 3).

### 3.2. Infection Study

Statistical analysis revealed a significant association between larval density and susceptibility to infection (*p* = 0.0010) and the interaction between density and food (*p* = 0.0224). Conversely, food availability alone did not show a significant influence on susceptibility to infection (*p* = 0.1708). Mosquitoes reared at a density of 180 individuals exhibited lower susceptibility to CHIKV infection compared to those raised at lower densities (Table 4). This density effect was primarily driven by the significantly lower infection rate observed in the 180-density treatment with high food availability (39.13%). Pairwise comparisons revealed significant differences in infection rates between the 60-density versus 180-density treatments (*p* = 0.0008).

**Figure 4:**
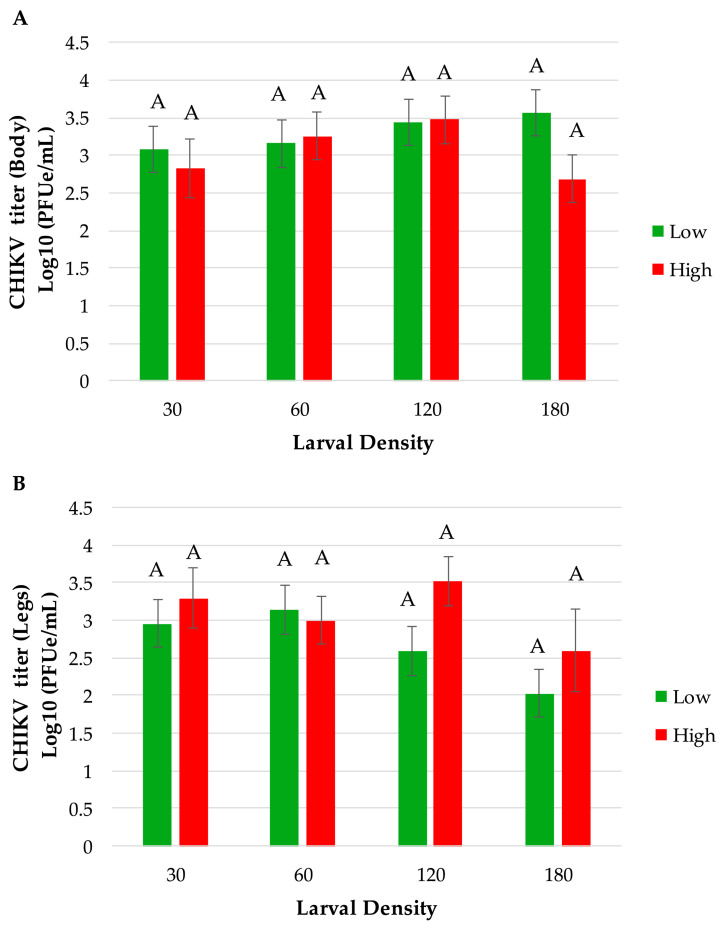
CHIKV viral titer in *Ae. albopictus* (**A**) bodies and (**B**) legs at 7 days post-infection. Statistical analysis was performed by a two-way ANOVA, bars represent means ± standard error of the means.

The percentage of mosquitoes exhibiting disseminated infection of CHIKV was measured by the number of females with infected bodies that had CHIKV RNA-positive legs. Overall, our results showed that mosquitoes’ viral dissemination was significantly affected by food effects and interaction between density and food (*p* = 0.0194 and *p* = 0.0473, respectively). We did not observe a significant effect by density alone (*p* = 0.0754). The significant food effect indicated that a higher percentage of mosquitoes exhibited disseminated infection (28% higher) derived from low-food conditions (45.62%) than from high-food conditions (32.78%). For the interaction, pairwise comparisons showed that mosquitoes from the 180 high food treatment had significantly lower disseminated infection (7.14%) compared to several other treatments, particularly 30 low (*p* = 0.0168) and 60 low (*p* = 0.0281). When analyzing the viral titer (expressed in log_10_ pfue/mL) in mosquito bodies and legs at seven days post-infection, ANOVA showed no significant effects between any of the treatment factors tested for food density and larval density (Table 5 and Figure 4). Although not significant, there is a trend for a lower viral titer in leg tissues among mosquitoes from the 180-larval-density treatment compared to lower-density treatments for low- and high-food conditions (Figure 4).

## 4. Discussion

Density-dependent interactions represent a critical regulatory influence in mosquito populations, exerting a substantial influence on immature aquatic stages and adult fitness. These interactions have significant implications for understanding vector biology, control strategies, and enhancing the probability of disease transmission risk [46]. Additionally, larval competition can affect vector–parasite interactions in adult mosquitoes [16,20].

The goal of our study was to investigate whether larval competition among Florida *Ae. albopictus* influences the vector competence for CHIKV. We hypothesized that nutrient limitation and larval competition decreases population growth correlates and increases susceptibility to infection and dissemination of CHIKV by adult mosquitoes. We evaluated this hypothesis using four treatment densities across a six-fold difference in the number of larvae and two food levels. For our assessment of population growth correlates, we showed that *Ae. albopictus* females and males were significantly affected by larval density. However, food treatment (except for males) and interaction between food and larval density were not significant. Development time appeared as the most important factor, followed by survivorship. For *Ae. albopictus* females and males higher-density treatments were associated with longer development times, while the highest rates of eclosion to adulthood were observed in low-density compared to high-density treatments.

These findings are similar to Braks and collaborators [13], where larval competition of Brazilian populations of *Ae. albopictus* and *Ae. aegypti* were measured under field conditions. In this study, the median time to adulthood for females of both species showed a comparable trend: median times of development for high-density treatments were greater than those of the lowest-density treatment. Our results were also in accordance with Bara et al. [18], with the goal of understanding how intraspecific larval competition affects dengue-2 virus (DENV-2) extrinsic incubation period and the mosquito’s overall ability to transmit the virus (vectorial capacity). By modeling the effects of larval competition using data from field and laboratory studies, they discovered that a twofold increase in larval competition resulted in significantly prolonged developmental time, fewer adult mosquitoes emerging, and smaller adult size. These findings align with previous studies demonstrating that intraspecific larval competition negatively impacts adult mosquito fitness at both the individual and population levels [47,48,49,50,51]. However, the competition had no effect on the extrinsic incubation period of DENV-2 in *Ae. albopictus.*

We observed associations between larval density, and interactive effects of larval density and food availability, on susceptibility to CHIKV infection. Our results also demonstrated that dissemination of CHIKV infection beyond the midgut to infect other mosquito tissues was altered by food availability alone and the interaction of food and larval density. A quantitative synthesis of mosquito infection studies, evaluating the influence of larval nutrition on adult mosquito vector competence for arboviruses (Ross River, Sindbis, dengue-2, La Crosse encephalitis, Murray Valley encephalitis, Japanese encephalitis, and West Nil viruses), showed that nutrient-deprived individuals exhibited higher rates of disseminated infection and transmission of arboviruses [46]. Interestingly, nutrient deprivation was not associated with changes in susceptibility to arbovirus infection. Thus, larval nutrition appeared to primarily alter the vector competence of adult mosquitoes for arboviruses by changes in the midgut escape (i.e., disseminated infection) and transmission barriers but had a negligible impact on midgut infection barriers [46]. Our observations of the directional effect of food availability on CHIKV disseminated infection and lack of an impact on susceptibility to CHIKV in *Ae. albopictus* corroborates these findings. Although other reviews have observed similar directional effects of larval nutrition on mosquito competence for arboviruses, alternative outcomes were also observed (e.g., no differences or lower infection rates) [52].

Contrary to our hypothesis, we observed lower susceptibility to infection and disseminated infection of CHIKV among *Ae. albopictus* adults derived from high-competition larval environments. These results differ from other studies [16,20], where competitively stressed *Ae. albopictus* females were more likely to have higher dengue virus and sindbis virus infection and dissemination than females from low-competition larval environments. In contrast, a study that reared larvae of *Ae. aegypti* from Thailand at densities of 50, 100, and 200 to produce three distinct size classes of adults (large, medium, and small, respectively) showed higher rates of disseminated infection of dengue-2 virus among larger mosquitoes (10.7%) than medium (5.6%) or small mosquitoes (5.7%) [53]. Along the same lines, Baqar et al. [54] observed that moderate larval competition significantly reduced susceptibility of *Cx. tritaeniorhynchus* to West Nile virus infection compared to low and high competition. Differences in findings between the current study and other studies raise important questions about the nature of larval competition in altering interactions between different arboviruses and mosquito species, as well as the need for standardized methods in assessments of vector competence for arboviruses in the context of environmental effects (biotic and abiotic factors).

Findings reported in other studies have found that different effects may occur with other arboviruses in areas where *Ae. albopictus* coexists with other mosquito species, such as *Aedes japonicus*, and *Culex pipiens* from Vanslembrouck [55]. Survival, development time, growth, and energy storage were measured in different European populations through density–response (intraspecific) and –replacement (interspecific) experiments. The authors explored how the outcome of larval interactions among these vectors is influenced by varying environmental conditions. Adult mosquitoes that survived the competitive larval phase were infected with specific viruses: West Nile virus for *Culex pipiens*, CHIKV for *Ae. albopictus*, and Japanese encephalitis virus for *Ae. japonicus*. It was demonstrated that interspecific competition resulted in larger mosquitoes, a higher arboviral infection rate, and an increased arboviral RNA titer. The observed increase in viral susceptibility following larval competition indicates a potential for higher arbovirus transmission rates in areas where these mosquito species coexist. Bevins [56] observed that interspecific competition with *Ae. albopictus* larvae led to the development of larger *Ae. triseriatus* females. These larger females were more susceptible to La Crosse virus infection compared to females from intraspecific experiments, suggesting that increased body size would provide more tissue for viral replication.

Differences in the observations of arbovirus infection rates among studies may be attributed to differences between specific vector–arbovirus and species interactions. While some field experiments attempt to replicate realistic conditions, it is crucial to acknowledge that variations in resource levels, types, frequencies, temperature, container type, larval density, and season may differentially influence the dynamics of larval competition among mosquito species [13,38,39,57] and consequently can affect the interactions of adults mosquitoes and viral pathogens [16,20].

As previously mentioned, our study must be considered in the context of our inability to analyze saliva infection to determine successful arbovirus transmission by bite to a vertebrate host. This limitation is due to the low number of *Ae. albopictus* females that fed on the honey provided in the Q-paper, which may provide new perspectives on data interpretation.

In summary, does nutrient availability and larval competition influence CHIKV infection in *Ae. albopictus*? Our findings indicate that food availability alone did not affect susceptibility to infection but did influence the percentage of mosquitoes exhibiting disseminated infection. Larval density and its interaction with food availability were significantly associated with increased susceptibility. These results highlight the influence of larval competition and availability of nutrients experienced during the immature stages in determining adult traits in *Ae. albopictus*, with important epidemiological implications for CHIKV transmission. Specifically, larval crowding associated with competition increased susceptibility to the virus but also depended on the availability of nutrition. Furthermore, varying ecological conditions can differentially influence mosquito–arbovirus interactions.

## Figures and Tables

**Table 1:** Analysis of population growth (lambda λ′) of *Ae. albopictus*. Least-squares means (±standard error) of estimated finite rate of increase for each treatment combination, food level treatment, and larval densities from two-way ANOVA. No statistically significant differences were detected among treatments (Tukey-adjusted *p* > 0.05).

Density	Food Level	Lambda λ′	Standard Error
30	High	1.465	0.1416
30	Low	1.396	0.1416
60	High	1.435	0.1416
60	Low	1.117	0.1416
120	High	1.110	0.1416
120	Low	1.345	0.1416
180	High	1.300	0.1416
180	Low	1.279	0.1416

**Table 2:** MANOVA results for main competitive treatment effects on female *Ae. albopictus* population growth measurements: survivorship (males and females), development time, and adult size. Significant effects are denoted in boldface *p*-values.

				Standardized Canonical Coefficients		
Factor	df	Pillai’s Trace	*p*	Survivorship	Development Time	Adult Size (Wing Length)
Larval density	9.81	1.02	**<0.0001**	0.46	1.84	−0.28
Food level	3.25	0.23	0.0775	0.93	−0.42	0.57
Larval density × food level	9.81	0.20	0.7306	0.47	1.43	−0.72

**Table 3:** MANOVA results for main competitive treatment effects on male *Ae. albopictus* population growth measurements: survivorship (males and females), development time, and adult size. Significant effects are denoted in boldface *p*-values.

				Standardized Canonical Coefficients		
Factor	df	Pillai’s Trace	*p*	Survivorship	Development Time	Adult Size (Wing Length)
Larval density	9.63	1.14	**0.0002**	1.32	2.24	0.90
Food level	3.19	0.34	**0.0195**	1.72	1.81	0.98
Larval density × food level	9.63	0.23	0.7966	0.77	0.83	−0.72

**Table 4:** CHIKV susceptibility to infection and disseminated infection in *Ae. albopictus*. Significant effects are denoted in boldface *p*-values.

Treatment Effect	Body Infection (%)	Estimate	Standard Error	*p*	Disseminated Infection (%)	Estimate	Standard Error	*p*
30 Low	76.92	−1.2040	0.3801	**0.0015**	45.16	0.1942	0.3609	0.5906
30 High	76.92	−1.2040	0.6583	0.0674	61.54	−0.4700	0.5701	0.4097
60 Low	82.69	−1.5640	0.3666	**<0.0001**	38.10	0.4855	0.3177	0.1265
60 High	79.66	−1.3652	0.3234	**<0.0001**	30.61	0.8183	0.3100	**0.0083**
120 Low	72.31	−0.9598	0.2772	**0.0005**	44.68	0.2136	0.2934	0.4666
120 High	73.33	−1.0116	0.4129	**0.0143**	31.82	0.7621	0.4577	0.0959
180 Low	71.74	−0.9316	0.3275	**0.0044**	54.55	−0.1823	0.3496	0.6020
180 High	39.13	0.4418	0.2467	0.0733	7.14	2.5649	0.7338	**0.0005**

**Table 5:** ANOVA for main effects on female *Ae. albopictus* CHIKV infected and disseminated infection.

Source	df	F-Value	*p*
**Body titer**			
Food	1	1.18	0.2936
Density	3	0.82	0.5036
Food × density	3	1.01	0.4158
**Leg titer**			
Food	1	2.66	0.1272
Density	3	1.63	0.2305
Food × density	3	0.95	0.4466

## Data Availability

Data are contained within the article.
